# Single Particle Differentiation through 2D Optical Fiber Trapping and Back-Scattered Signal Statistical Analysis: An Exploratory Approach

**DOI:** 10.3390/s18030710

**Published:** 2018-02-27

**Authors:** Joana S. Paiva, Rita S. R. Ribeiro, João P. S. Cunha, Carla C. Rosa, Pedro A. S. Jorge

**Affiliations:** 1 INESC TEC-INESC Technology and Science, 4200 Porto, Portugal; Arsr@inescporto.pt (R.S.R.R.); jpcunha@fe.up.pt (J.P.S.C.); ccrosa@fc.up.pt (C.C.R.); pedro.jorge@fc.up.pt (P.A.S.J.); 2 Physics and Astronomy Department, Faculty of Sciences, University of Porto, 4169-007 Porto, Portugal; 3 Faculty of Engineering, University of Porto, 4200 Porto, Portugal

**Keywords:** polymeric optical lenses, optical fibers, micromanipulation, back-scattering, signal processing, features dimensionality reduction techniques, Linear Discriminant Analysis, particles sorting and differentiation

## Abstract

Recent trends on microbiology point out the urge to develop optical micro-tools with multifunctionalities such as simultaneous manipulation and sensing. Considering that miniaturization has been recognized as one of the most important paradigms of emerging sensing biotechnologies, optical fiber tools, including Optical Fiber Tweezers (OFTs), are suitable candidates for developing multifunctional small sensors for Medicine and Biology. OFTs are flexible and versatile optotools based on fibers with one extremity patterned to form a micro-lens. These are able to focus laser beams and exert forces onto microparticles strong enough (piconewtons) to trap and manipulate them. In this paper, through an exploratory analysis of a 45 features set, including time and frequency-domain parameters of the back-scattered signal of particles trapped by a polymeric lens, we created a novel single feature able to differentiate synthetic particles (PMMA and Polystyrene) from living yeasts cells. This single statistical feature can be useful for the development of label-free hybrid optical fiber sensors with applications in infectious diseases detection or cells sorting. It can also contribute, by revealing the most significant information that can be extracted from the scattered signal, to the development of a simpler method for particles characterization (in terms of composition, heterogeneity degree) than existent technologies.

## 1. Introduction

Recent trends on healthcare or microbiology industry point out the urge to develop micro-tools with a variety of functionalities such as simultaneous manipulation and sensing [1,2,3,4,5]. Considering that medical and consumer technology are converging to a novel paradigm of miniaturization to the micro and nano-level, optical fiber tools (e.g., Optical Tweezers-OT) are suitable platforms to host multifunctional small sensors with applications in Medicine and Biology [1]. Additionally, taking into consideration the flexibility, small size and chemical inertness of optical fibers, an optotool able to simultaneously guide light, trap, manipulate and differentiate the type of trapped particle can be an outstanding contribution for areas such as medical diagnosis [5,6], air/water pollution monitoring or food industry. Nevertheless, the development of such hybrid opto-tools would never be possible without the improvements to optical trapping and manipulation that light driven tools as OTs have seen during the last 10 years [6,7].

The optical trapping effect was first demonstrated in 1970, by Ashkin et al. [8], through the stable trapping of a microparticle due to the radiation pressure exerted by two counter propagated laser beams [8]. Early on, OTs were considered flexible and versatile micromanipulation tools for a wide range of application fields including Biology, Photonics, Microrheology, Biomedicine and Quantum Physics [7,9,10]. Due to their ability to exert piconewton forces, they can be used to trap, manipulate and study micro-sized objects, including synthetic particles, cells or even cellular organelles [1,7,8]. However, the most conventional OT setups (COTs) are based on complex and expensive configurations-including bulky laboratory equipment such as inverted microscopes-, being associated with focusing difficulties in turbid media and thick samples (e.g., biological cells and tissues) [1,7].

Optical Fiber Tweezers (OFTs), which consist in optical fibers with the extremity patterned or modified to form a micro lens to tightly focus the propagating laser beam, have been considered a valuable alternative to COTs [7,11,12]. In fact, the momentum transfer between the radiation field that emerges from the fiber tip and the scattered particle, under certain conditions, is enough for trapping the scatterer, in a contactless and minimally invasive manner [1,7,11,12]. Although the development of OTs continues to be a growing field, a limited number of research teams are currently working with OFTs [10,13,14,15,16,17,18]. The number of explored operations that OFTs are able to perform is still limited, being these, in general, uniquely focused on trapping [1,7].

Alternatively to OFTs for simultaneous trapping and sensing, plasmonic tweezers, by providing particles trapping abilities and for being sensitive to tiny changes of refractive index in the surrounding material, have been also explored for developing optical tools that, besides trapping, can provide some information about the trapped object [19,20,21]. However, according to some studies, the high refractive index sensitivity of plasmonic tweezers can have a destructive impact whenever the presence of the trapped scatterer in the trapping site reduces the electromagnetic beam local intensity and/or field gradient, weakening the trapping phenomena [19,22]. Additionally, the majority of plasmonic tweezers are composed by bulky microscope equipment, similarly to COTs [19,23,24]. However, flexible configurations based on optical fibers with plasmonic properties have been purposed for simultaneous trapping and sensing [25,26].

Apart from the available opto-tools for manipulation and trapping, the existent optical fiber sensors for particles detection have also limitations. They are also based on complex configurations [27,28], require expensive microfabrication methods [29] or fiber tip functionalization techniques using expensive dyes, antibodies or organic functional groups [30]. Additionally, they do not allow particle trapping. Even the alternatives to optical fiber sensors in terms of label-free particles detection and differentiation (e.g., Raman Spectroscopy, Fourier-Transform Infrared Spectroscopy) are, in their majority, complex, non-portable and expensive [3]. Thus, the development of a hybrid miniaturized optical device in which the tip of an optical fiber is able to simultaneously guide light, trap and sense micro-sized bioparticles could be a great progress for bioanalitics [31]. According to conventional approaches, since the quantity to be measured modulates the intensity, phase, polarization state or wavelength of the light in the fiber, only a single parameter is usually interrogated for sensing purposes [32]. In fact, there are much more relevant parameters that can be extracted from the sensor output signal. However, their simultaneous analysis considering a features space with more than three dimensions can be a hard task.

The amount of light scattered by a particle has been considered a gold-standard technique for simple cell/particle characterization, given its dependence with crucial scatterer characteristics such as particle diameter, refractive index, shape/geometry, composition and content type (synthetic, biologic) [33,34,35]. Different cells or cellular organelles are often distinct in terms of their refractive index values due to the types of proteins expressed and intracellular cargo differences between them [35]. Infection by parasites could also cause changes in the refractive index distribution in certain cells, as for example Red Blood Cells (RBCs) infected by *Malaria* [5,36].

With this in mind, we report in the present paper a study on the following research questions: (1) does frequency- and/or time-domain features of back-scattered signal from trapped particles contain enough information to differentiate distinct types of scatterers? (2) If yes, is there a single feature, composed from the studied more relevant frequency- and time-domain parameters, that reflects the above mentioned differentiation? To answer to our research questions, we analyzed 9 time-domain (7 time-domain statistics and 2 time-domain histogram features) and 36 frequency-domain features of the back-scattered signal. Once it could be a difficult task for a sensor reading system to directly aggregate the relevant information provided from 45 distinct parameters for differentiating samples into different types, we applied a feature extraction technique-the Linear Discriminant Analysis (LDA)-to project all the important information into a single feature, much less demanding and easier to analyze. This process is frequently used for features dimensionality reduction in Data Mining and Multivariate Data Analysis, to eliminate noisy and redundant information, reduce computational complexity and improve decision function generalization ability. This novel adimensional feature was tested to differentiate (with statistical significance) four different conditions corresponding to the identification of different types of trapped particles: “PMMA particle trapped”, “Polystyrene particle trapped”, “living yeast cell trapped” and “no particle trapped” in de-ionized water, as well as all the possible parwise combinations between these four classes. All the particles used in this study had similar sizes in order to exclude the hypothesis of particles differentiation due to the influence of the target size on the amount of light scattered.

To the best of our knowledge, this is the first time (in addition to a study conducted also by our lab [1]) that simultaneous optical trapping and short-term back-scattered signal analysis by means of a polymeric microlens is addressed for microparticles differentiation. The exploratory analysis conducted in this study as well as a novel single feature could be extremely useful for simultaneous microparticles immobilization and classification. The selection of the most relevant attributes for differentiating the four classes and the determination of the contribution weight of each original feature into the final one can reveal which particle parameters provide information about its type (synthetic versus biologic), composition, size, heterogeneity degree and new insights about scattering. Additionally, it could have applications in healthcare for rapid clinical diagnosis (e.g., detection of a circulating *Malaria*-infected blood cell).

## 2. Materials and Methods

### 2.1. Optical Trapping Experiments

In this section, the fabrication method of the spherical lenses on top of optical fibers (see Section 2.1.1) as well as the optical manipulation setup built for trapping and back-scattered signal acquisition (see Section 2.1.2) are described. The optical fiber selected to work with was a 980 nm *Thorlabs* single mode fiber (SMF) (Thorlabs SM 980-5.8-125, Thorlabs, Newton, NJ, USA).

#### 2.1.1. Fabrication of the Polymeric Lens for Optical Trapping

**Figure 1:**
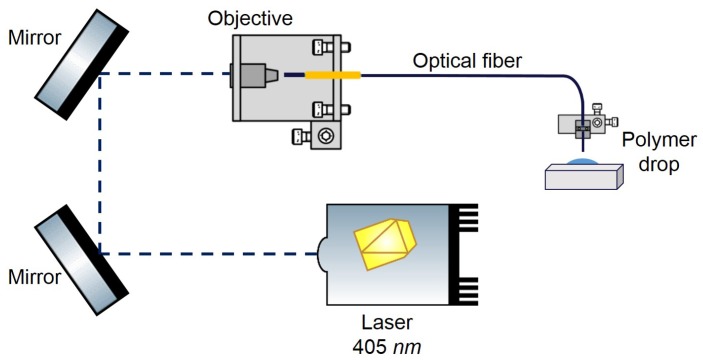
Scheme of the optical setup used to couple the 405 nm laser used to polymerize the micro structures on the optical fiber tips.

**Figure 2:**
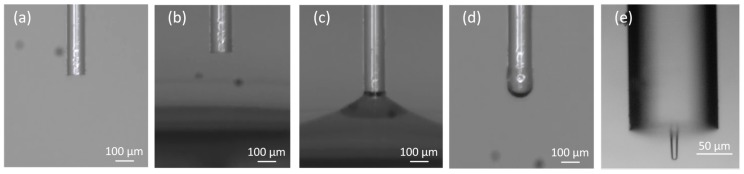
Fabrication process stages of the polymeric spherical tips on top of optical fibers used in this study (**a**–**e**).

The polymeric trapping lens used in this study was fabricated using a guided photo-polymerization method that was developed by Soppera et al [37] in collaboration with our lab [7,12,38,39,40]. Polymeric OFTs fabricated using this process and able to successfully trap in 2D both PMMA and polystyrene beads, yeast and plant cells were already obtained in our laboratory [7,12,37,38,39,40,41]. This process is mainly based on the assemble of cross-linked polymeric structures through monomers linking, triggered by light of a specific wavelength [1,7,37]—please see Figure 1 for a schematics of the lenses fabrication setup. In this particular case, the pentaerythriol triacrylate (PETIA)—n=1.48—and Bis(2,4,6-trimethylbenzoyl)-phenylphosphineoxide, commercially known as Irgacure 819, were used as monomer and photo-initiator in the photo-polymerization reaction, respectively. Since the Irgacure 819 is sensitive to wavelength values between 375 and 450 nm, a violet diode 405 nm laser (LuxX cw, 60 mW, Omicron) was used to trigger the polymeric cross-linking chain reaction [7]. This guided photo-polymerization method is composed by the following steps: at first an optical fiber is cleaved at its extremities and one of them is positioned vertically in a moving stage (Figure 2a), while the 405 nm laser light is aligned to be injected, in the distal end. Then, the optical fiber extremity is slowly dipped into the solution with the monomer and initiator substances (Figure 2b,c). After slow removal of the fiber tip from the solution, a polymer drop is formed in its extremity (Figure 2d), which is then irradiated through the core and consequently cured. The last step consists in washing out the remaining liquid from the polymeric tip using ethanol (Figure 2e) [1,7]. This method is characterized by a self-assembly effect, since during polymerization, the refractive index of the growing tip increases, which creates a self-guiding effect. This effect then prevents the radiation from scattering in the remaining of the drop. The refractive index of the cured polymeric tip is about 1.52. However, it is important to mention that, in this process, when dealing with multimode fibers, the final geometry of the fiber tip depends on the fiber mode that is excited during the irradiation process. Thus, since the fiber used in this study behaves as a multimode waveguide at a wavelength of 405 nm, the fiber mode chosen to be excited was the fundamental one, in order to obtain a spherical tip whose diameter was perfectly matched to the fiber core [1,7]—please see Figure 2e. However, besides the excited fiber mode, there are other parameters that influence the geometry of the final lens, such as exposure time, laser intensity, ratio between the amount of monomer and photo-initiator used for polymerization, irradiation exposure time, and the length/curvature radius of the monomer/initiator solution drop [1]. Since those were already optimized to attain the best particle trapping performance as possible [7,12,37,38,39,40,41], a polymer mixture containing 0.2% in weight of Irgacure 819, a laser exposure time of 60 s and a power of, at least 20 μW at 405 nm, were used in the photo-polymerization process. The visual aspect of the fabricated tip used in the trapping experiments is provided in Figure 2e.

#### 2.1.2. Optical Trapping and Particles Sensing Setup

**Figure 3:**
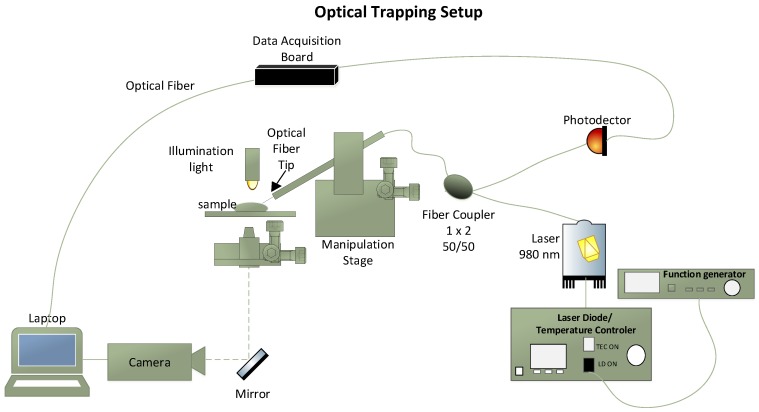
Optical manipulation setup.

The experimental setup used to manipulate the polystyrene, PMMA microparticles and living yeast cells using the fabricated fiber tweezers is presented in Figure 3. It is composed by a home made inverted microscope containing a 20× objective, connected to an image acquisition system, a 4-axis motorized micromanipulator (x,y,z and angular) to handle the fabricated optical fiber tip and a back-scattered light acquisition module. A pigtailed 980 nm laser (500 mW, Lumics, ref. LU0980M500, Berlin, Germany) and the back-scattered signal acquisition module were connected to a 50/50 980 nm fiber coupler with a 1 × 2 topology. The optical fiber tip was then spliced to the output of the optical coupler and inserted into a metallic capillary controlled by the motorized micromanipulator. The capillary was tilted at 50°, since trapping phenomena is only possible for fiber tip inclination angles >30° [41]. This configuration allowed both laser light guidance to the optical fiber tip through the optical fiber and the acquisition of the back-scattered signal through a photodetector (PDA 36A-EC, Thorlabs, Newton, NJ, USA)—please see Figure 3. The image acquisition system was composed by a CMOS camera (EO-2018C, Edmund Optics, Barrington, NJ, USA) connected to a laptop. In addition to the photodetector, the back-scattered signal acquisition module was also composed by a data acquisition board (DAQ from National Instruments, TX, USA), which was connected to the photodector for transmitting the acquired signal to the laptop. A function generator was also included in the trapping setup for modulating the laser diode 980 nm signal. A drop of de-ionized water containing the microparticles was then placed over a glass coverslip over the inverted microscope setup. The fiber with the lensed tip on its extremity was inserted into this sample, while the immersed micro tip as well manipulated microparticles were visualized using the CMOS camera. The output laser diode power was set to ≈10 mW during the experiment, a trade-off value to avoid damage to biological cells (yeast cells) and, at the same time, to ensure a stable trapping [41].

### 2.2. Particles Trapping and Sensing Using the Polymeric Lens

Three types of aqueous solutions containing different type of particles in suspension were used in this experiment (please see Table 1).

**Figure 4:**
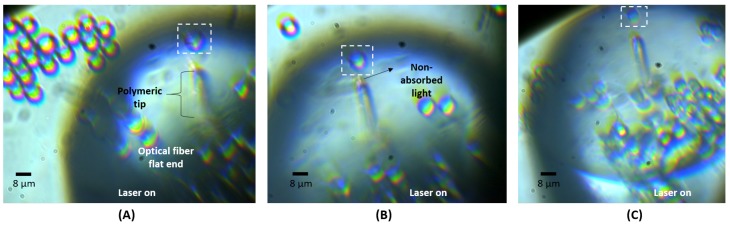
Video frames showing (**A**) a polystyrene; (**B**) a PMMA microparticle and (**C**) a living yeast cell stably trapped in front of the fabricated polymeric tip embedded in de-ionized water.

For each solution, a simple assay was carried out where a drop was placed over a glass coverslip and the optical fiber tip inserted into the sample. After the immersed lensed tip carefully positioned, with the help of the mounted imaging system, in front of an isolated particle, the laser was turned on (@980 nm; optical fiber tip output power of 10 mW). The laser light was modulated with a sinusoidal signal (frequency 1 KHz), to allow synchronous detection of the back-scattered signal in a frequency band with lower electronic noise. Once the particle was stably trapped as illustrated in Figure 4, the back-scattered signal was acquired according to the procedure detailed in point Section 2.2.1. Short-term segments of signal were also acquired for the case of no particle in front of the tip, representing the class “No particle trapped”.

#### 2.2.1. Back-Scattered Signal Acquisition and Processing Steps

Back-scattered signal was acquired through a photodetector (PDA 36A-EC, Thorlabs) connected to a Analog-to-Digital converter (National Instruments DAQ) at a sampling rate of 5 kHz. A MATLAB 2015a^®^ custom-built script was used for both signal acquisition and processing. Signal Processing and Statistics toolboxes from Matlab^®^ were used during signal processing and analysis steps.

**Figure 5:**
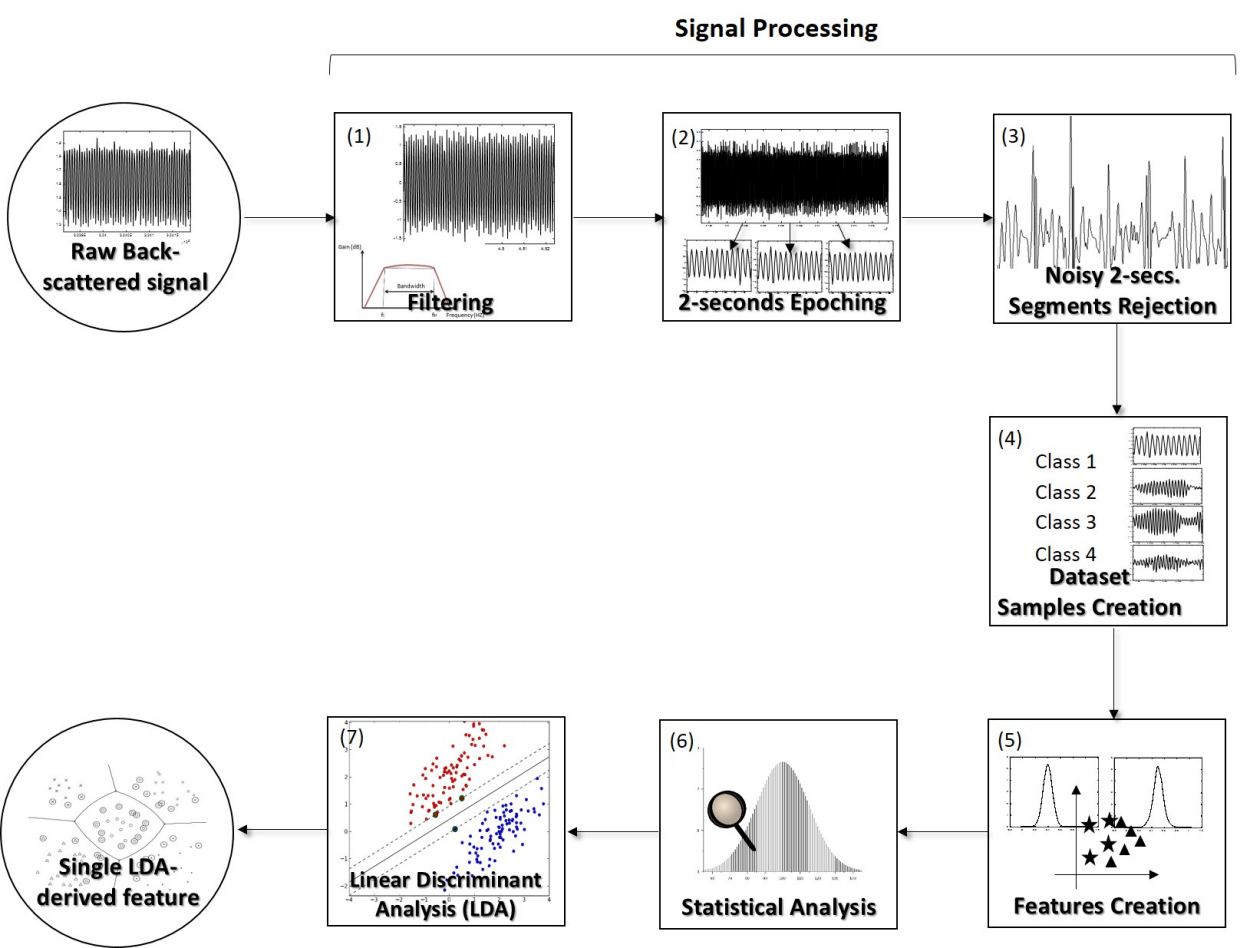
Scheme explaining all the back-scattered signal processing and analysis steps. (**1**) At first each 120-s whole acquisition was filtered using a second-order 500 Hz Butterworth high-pass filter. (**2**) Then, each entire acquisition corresponding to each particle from each class was split in 2 s signal epochs. (**3**) In order to remove the noisy portions, each signal portion was *z-scored* and discarded if one of its values exceeded |zscore|>5. (**4**) After these steps, it was possible to obtain a dataset with 2 s signal portions with a reasonable SNR for the particles type differentiation to be possible. Class 1—“no particle trapped”; Class 2—“PMMA particle trapped”; Class 3—“Polystyrene particle trapped”; Class 4—“Living yeast cell trapped”. (**5**) Then, the 45 signal-derived features to be evaluated were computed. (**6**) Those were studied using statistics. (**7**) The most relevant information present in the original features set was gathered into a single one, using the dimensionality reduction technique Linear Discriminant Analysis (LDA).

After each acquisition of 120 s of back-scattered signal per particle, the original signal was passed through some processing steps, which will be described in the following paragraph. A total of 7920 s of back-scattered signal was acquired considering all the classes and particles analyzed—please consult Table 2 for more details about the dataset. “No particle" signal acquisitions were performed by moving the polymeric tip into an empty area, where, despite the laser being turned on, no particle was trapped. 120-s “no particle” acquisitions were obtained for sixteen times, in 16 different media locations. The “No particle” condition was treated in the same way as a type of microparticle. The inclusion of such class (of “no particle trapped”) in this problem could be relevant to find the best parameter or set of parameters to continuously verify if a given particle was trapped or not. After signal processing, our dataset was composed of back-scattered signal portions of 2 s (each sample of the dataset). After removing the noisy samples in the artifact rejection stage, a set of 45 features characterizing each 2 s signal portion was created and its potential to differentiate the classes “Class 1: no particle trapped”, “Class 2: PMMA particle trapped”, “Class 3: Polystyrene particle trapped” or “Class 4: Living yeast cell trapped” was individually (feature by feature) evaluated in the statistical analysis stage. Please consult Section 2.2.2 for a detailed characterization of the signal-derived features analyzed. Considering that the inclusion of 45 features in the differentiation task would be difficult to design a reading system or a decision function that takes into account so many attributes for particles sensing, a features dimensionality reduction method was applied to build a single feature. The Linear Discriminant Analysis (LDA)—please consult point Section 2.2.4 for more details about this method-was used to create such feature and its statistically relevance was also evaluated. In Figure 5 is presented a scheme summarizing all the steps conducted for data analysis.

**Figure 6:**
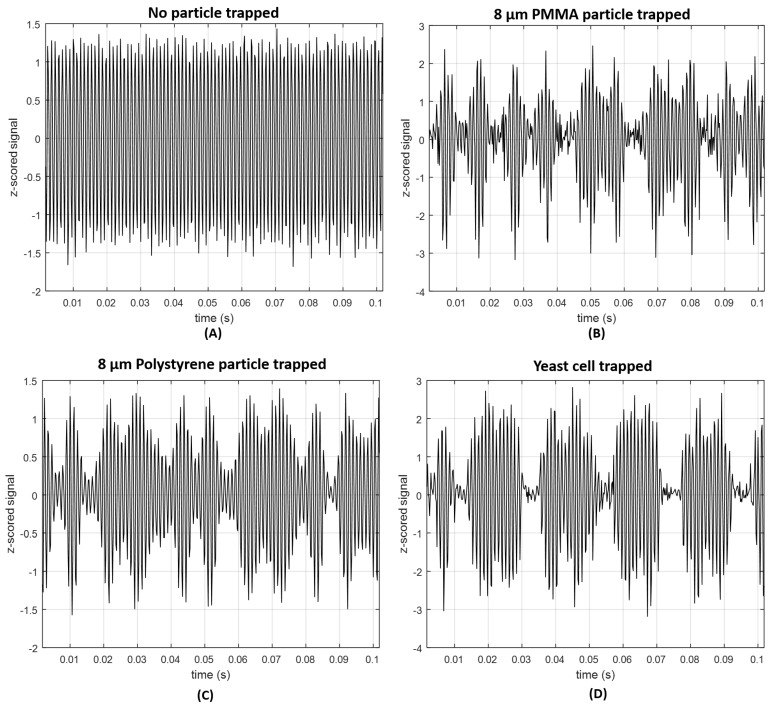
Portion of back-scattered signals obtained after signal processing steps for (**A**) “no particle trapped”, (**B**) “PMMA particle trapped”; (**C**) “Polystyrene particle trapped" and (**D**) “Living yeast cell trapped”.

During signal processing, the signal was at first filtered, using a second-order 500 Hz Butterworth high-pass filter, since the input irradiation laser was modulated using a 1 kHz sinusoidal signal, and to remove noisy low-frequency components of the acquired signal (e.g., 50 Hz electrical grid component). This type of filter was already successfully applied to this type of signal also to differentiate between aggregates and isolated particles from different sizes in a previous study of our group [1]. Then, the entire signal acquired for each particle and condition was split into epochs of 2 s. The *z-score* of each 2 s signal portion was computed in order to remove noisy signal epochs. Independently of the type of features used in this kind of problems, it is important that their raw signals have as higher signal-to-noise ratio (SNR) as possible [44,45,46]. 2 s *z-scored* signal portions which, in magnitude, exceeded the threshold value of 5 were therefore discarded. Sketches of signal portions for each one of the four classes are provided in Figure 6.

#### 2.2.2. Particles Differentiation Using Back-Scattered Trapping Signal

The paragraphs below describe the 45 features/attributes set extracted from the back-scattered signal of a trapped particle used to differentiate its type.

#### Features

The choice of the features or attributes for characterizing a specific class is an important step in Multivariate Data Analysis [44]. They must be as relevant as possible and should not be redundant relatively to each other [44,47,48]. Features highly correlated could compromise differentiation ability [48]. In this particular problem, we chose a total of 45 features to characterize each class that could be separated in two main types: time-domain and frequency-domain features. The first set can be divided into two subsets: time-domain statistics and time-domain histogram-derived features. The frequency-domain set is also divided into two groups: Discrete Cosine Transform (DCT)-derived features [49] and Wavelet features [50]. The 45 features considered are summarized in Table 3.

Several studies based on the identification of targets type through the scattered signal including underwater fish species recognition [51,52,53,54,55,56,57,58] or objects identification in the surrounding environment (in air, water, etc) [59] use time-domain statistical characteristics, Wavelets, matching pursuit methods or features correlated with the energy or shape of echoes [60]. Considering the lack of studies involving the analysis of the back-scattered signal provided from a trapped particle acquired through the optical fiber, the features explored here were selected based on previous studies about object recognition through scattering signals acquired using photodetectors or other kind of “event counter” equipment.

#### Time Doman-Derived Features

Time domain statistics parameters such as Mean (M), Standard Deviation (SD), Root Mean Square (RMS), Skewness (Skew), Kurtosis (Kurt), Interquartile Range (IQR) and Entropy (E) were used, given its adequacy in differentiating types of periodic signals providing from different origins, from synthetic to biological sources [1,51,61,62]. Statistical time-domain parameters have been used to differentiate pathological from healthy electrocardiogram (ECG) and pulse waveform samples [11,45,61,62], to identify tumor cell clusters in cell lines [63], to differentiate between aggregates and isolated particles [1] or to identify different objects also through the back-scattered signal in underwater conditions [51,52,53]. High order moment-based features such as SD, Kurtosis and Skewness have been considered robust to aspects such as size or shape [51]. The Skewness reflects the distribution symmetry degree, and Kurtosis quantifies whether the shape of the data distribution matches the Gaussian distribution [64,65]. Both have been widely used in several signal processing approaches, for quantifying how far, in statistical terms, the evaluated sample distribution is from a normal one [64,66]. Once the IQR is a widely used variability measure [62,65] it was also included in this set.

The potential of time-domain histogram-derived features to successfully differentiate the four classes were also evaluated. Two parameters were therefore extracted from the histogram of each 2 s back-scattered time-domain signal portions, representing how frequently each time-domain value was recorded along each signal portion. According to several studies, the Nakagami distribution can adequately describe the back-scattered echo in statistical terms [67]. This type of statistical distribution is a simplified, straightforward and more general version of the Rayleigh or K distributions, developed to reduce the analytical complexity involved with these two and to encompass different scattering conditions [67]. It is robust to the presence of an ensemble of scatterers with varying number densities, varying cross sections and to the presence or absence of regularly spaced scatterers. It has been widely used for biological tissues differentiation (i.e., between tumoral and healthy human tissue) through back-scattered light [68,69]. The Probability Density Function (PDF) of the Nakagami distribution is given by the following equation [67]:

(1) $$PDF\left(x;\mu ,\omega \right)=2{\left(\frac{\mu }{\omega }\right)}^{\mu }\frac{1}{\gamma (\mu )}x^{\left(2\mu -1\right)}\exp^{\frac{-\mu x^{2}}{\omega }}\;,$$

in which μ is the shape parameter and ω>0 the scale parameter, for x>0. Considering that *x* is the PDF of the time-domain histogram of each back-scattered signal portion, we extracted the parameters $\mu_{Nakagami}$ and $\omega_{Nakagami}$ that better fit the approximation of our distribution to the Nakagami distribution.

#### Frequency Doman-Derived Features

Regarding the frequency-domain analysis of the back-scattered signal, two types of features were analyzed: a set of parameters based on the Discrete Cosine Transform (DCT) and another set derived from Wavelet analysis. Considering that our goal was to extract meaningful features from short signal portions (2 s), the widely known Fast-Fourier Transform (FFT) method was not suitable for this particular problem. Although the acquisition sampling rate was determined to comply with the Nyquist theorem, considering the short duration of the analyzed signal portions, the spectrum of a radiated signal consists of two different types of contribution: a continuous contribution broadband component and a discontinuous narrowband component [70]. The back-scattered signal is a mixture of these two types of signals [52,70]. In order to collect the two types of information, we decided to apply the Discrete Cosine Transform (DCT) [71] to each signal portion, rather than the FFT. The DCT, in contrast to the FFT, is also able to capture minimal periodicities of the signal, without injecting high-frequency artifacts in the transformed data. Besides being more adequate to short signals, it is also highly attractive for this type of problems which require to differentiate target classes, because DCT coefficients are uncorrelated. Thus, they can be used as suitable features for characterizing each class [65]. Additionally, the DCT is able to embed most of the signal energy into a small number of coefficients. The first *n* coefficients of the DCT of the scattering echo signal are defined by the following equation [60]:

(2) $$E_{i}^{DCT}\left[l\right]=\sum_{k=0}^{N-1}\varepsilon_{i}\left[k\right]\cos\left[\frac{\pi l(2k+1)}{2N}\right],\;for\;l=1,\ldots ,n\;,$$

in which $\varepsilon_{i}$ is signal envelope estimated using the Hilbert transform. By sorting the DCT coefficients from the highest to the lowest value of magnitude and obtaining the following vector:

(3) $$y_{i}={\left(E_{i}^{DCT},\ldots ,E_{i}^{DCT}\left[l^{n}\right]\right)}^{T}\;,$$

in which $E_{i}^{DCT}\left[l^{1}\right]$ represents the highest DCT coefficient in magnitude, it is possible to determine the percentage of the total amount of the signal energy that each coefficient represents. Each percentage value is obtained by dividing the norm of the vector formed by the first till the *n*th coefficient by the norm of the vector composed by all the *n* coefficients. As an example, the percentage (P) of the total original signal energy that the three first coefficients of the vector defined in (3) are able to capture is given by the following expression [55,60]:

(4) $$\begin{array}{c} P=\frac{\left|\vec{v}\right|}{\left|\vec{v_{t}}\right|}*100\;,\\ \vec{v}=\left[E_{i}^{DCT}\left[l^{1}\right],E_{i}^{DCT}\left[l^{2}\right],E_{i}^{DCT}\left[l^{3}\right]\right]\;,\\ \vec{v_{t}}=\left[E_{i}^{DCT}\left[l^{1}\right],\ldots ,E_{i}^{DCT}\left[l^{n}\right]\right]\;, \end{array}$$

in which $\vec{v}$ is a 3 dimensional vector and $\vec{v_{t}}$ a *n* dimensional vector. The following features were evaluated from DCT: the number of coefficients needed to represent about 98% of the total energy of the original signal ($N_{DCT}$), the first 20 DCT coefficients extracted from the vector defined in (3), the Area Under the Curve (AUC) of the DCT spectrum for all the frequencies (from 0 to 2.5 kHz) ($AUC_{DCT}$), the maximum amplitude of the DCT spectrum ($Peak_{DCT}$) and the signal power spectrum obtained through the DCT considering all the values within the frequency range analyzed (from 0 to to 2.5 kHz) ($P_{DCT}$).

Some parameters based on the information extracted from Wavelet analysis of the original signal were also evaluated. Signal decomposition by Wavelets is a signal processing method widely used that allows frequency subband decomposition with the possibility to retain relevant temporal-spectral properties from the original signal [72]. The concept behind Wavelet analysis is that, for a given signal, a pair of low-pass and high-pass filters is used to yield two sequences capturing information from different frequency subbands of the original signal. These sequences are then subsampled by a factor of two, leading to one level of signal decomposition by Wavelets. This process can be therefore repeated for small partitions of the frequency spectrum for resolving different subtle features while localizing temporal information [55,72]. Thus, using Wavelet packet decomposition it is possible to extract, in each subband, certain tonal information of the original signal depending on the frequency range and content of the back-scattered signal [55,72]. For Wavelet packet signal decomposition, it is necessary to choose a suitable mother Wavelet, that will be used as a prototype to be compared with the original signal and extract frequency subbands information [55,62,72]. Generally, a mother Wavelet similar in shape to the original signal is considered appropriate [72]. Two mother Wavelets-*Haar* and Daubechies (*Db10*)-were selected to characterize the back-scattered signal portions, considering their simplicity and the fact that they were already successfully used to decompose back-scattered signals in underwater scenarios [60,73]. Six features for each type of mother Wavelet based on the relative power of the Wavelet packet-derived reconstructed signal (one to six levels) were evaluated, totaling 12 features [60,62,73].

#### 2.2.3. Statistical Analysis

Non parametric statistical tests were applied, due to the fact that some of the features analyzed failed to be normal distributed (Shapiro-Wilk Normality Test). Statistical evaluation was conducted using the Statistics Toolbox from MATLAB R2015a^®^. All values for each feature representing 2 s signal portions and belonging to the same particle were averaged in a way that vectors containing values for statistical comparisons were arranged in a [1 × totalnr.particles] scheme, for each condition evaluated (“No particle”, “PMMA particle”; “PS particle” and “yeast cell”).

The Kruskal-Wallis test was applied to each one of the 45 features analyzed in order to identify the ones suitable to differentiate the four classes considered (Class 1: “No Particle”; Class 2: “PMMA particle”; Class 3: “PS particle”; Class 4: “Living yeast cell”). A post-hoc parwise analysis was also conducted for each one of the variables analyzed after applying the Kruskal-Wallis test for 4 conditions, as indicated by Pallant [74]. The Mann-Whitney test (2 conditions) was then performed to evaluate the differentiation ability of each feature in a parwise manner (considering the six possible combinations between two classes-Class 1 vs. Class 2; Class 1 vs. Class 3; Class 1 vs. Class 4; Class 2 vs. Class 3; Class 2 vs. Class 4; Class 3 vs. Class 4) [74]. The potential for differentiating both in a 4 classes or parwise manner of the novel single feature created using the LDA technique was also evaluated using the Kruskal-Wallis (4 conditions) and Mann-Whitney (2 conditions) statistical tests, respectively. The statistical significance level of 0.05 was considered for all the statistical tests conducted [74].

#### 2.2.4. Features Dimensionality Reduction Using Linear Discriminant Analysis (LDA)

As already mentioned above, we must only include features that contribute with relevant information in a multiclass differentiation problem [75]. The elimination of highly correlated features (also called dimensions in Multivariate problems) or redundant information will reduce the computational cost and simplify the decision task [44,75]. For this reason, we applied a features extraction technique-the Linear Discriminant Analysis (LDA)-in order to include in a single feature the most significant information from the original set of 45 features. Despite of this novel single feature being adimensional, it is possible to obtain the contribution weights of each original feature in the calculation of this “optimal” feature. Such information can be easily calculated, since the novel feature(s) generated by LDA result(s) from linear combination(s) of the original ones [44,75].

**Figure 7:**
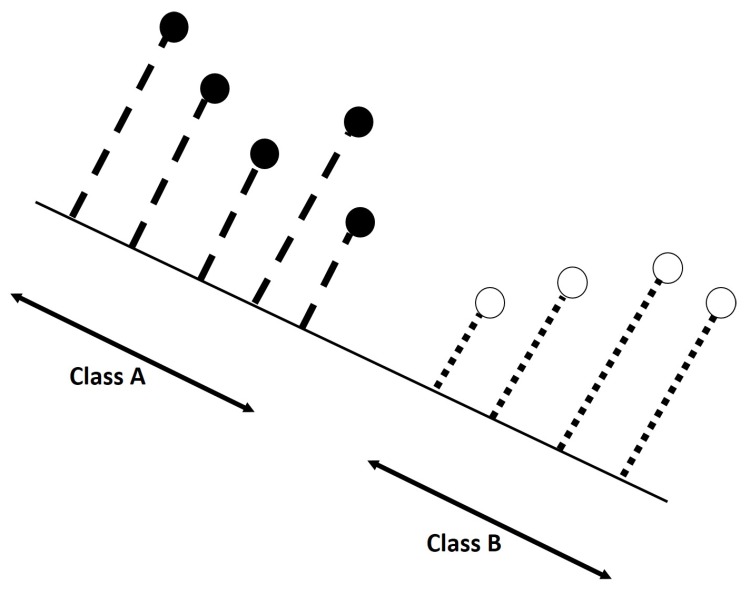
Scheme explaining the main objective behind LDA. Data samples in 2D (N=2, two features) are projected in a lower dimensions space (a line, 1D). The line must to be chosen in way that the projection maximizes the “separability” of the projected samples.

Considering the *N*-dimensional features space (in which *N* represents the number of features), the LDA tries to find the projection hyperplane that minimizes the interclass variance and maximizes the distance between the projected features means of the classes [44,75,76]. These two main aims can be achieved by solving an eigenvalue problem with the corresponding eigenvector defining the hyperplane of interest [76]. In simpler terms, the LDA consists in determining a subspace of lower dimension, in which the data points of the original problem are “separable” (in terms of statistical measures of mean value and variance)—please see Figure 7. One of the advantages that characterize LDA is that the solution can be found by solving a generalized eigenvalue system. However, LDA may not be suitable when the classes are not linearly separable [76].

Let $x_{1},\ldots ,x_{p}$ be a set of *p* data samples which belong to two different classes A and B. If we consider the sample means for each class as [76]:

(5) $${\bar{x}}_{A}=\frac{1}{N_{A}}\sum_{x\in A}x,\;{\bar{x}}_{B}=\frac{1}{N_{B}}\sum_{x\in B}x\;,$$

in which $N_{A}$ and $N_{B}$ are the total number of samples in A and B, respectively; we can define the positive semidefinite scatter matrices described by Equations [76]:

(6) $$S_{A}=\sum_{x\in A}\left(x-{\bar{x}}_{A}\right){\left(x-{\bar{x}}_{A}\right)}^{T},\;S_{B}=\sum_{x\in B}\left(x-{\bar{x}}_{B}\right){\left(x-{\bar{x}}_{B}\right)}^{T}\;,$$

in which each of these matrices represent the sample variability in each class. Theoretically, the aim of LDA is to find a hyperplane (defined by the vector ϕ), according to which, if the data samples were projected, their variance would be minimal, which corresponds having [76]:

(7) $$\min_{\phi }\left(\phi^{T}S_{A}\phi +\phi^{T}S_{B}\phi \right)=\min_{\phi }\phi^{T}\left(S_{A}+S_{B}\right)\phi =\min_{\phi }\phi^{T}S\phi \;,$$

where $S=S_{A}+S_{B}$ and the scatter matrix between the two classes is defined by [76]:

(8) $$S_{AB}=\left({\bar{x}}_{A}-{\bar{x}}_{B}\right){\left({\bar{x}}_{A}-{\bar{x}}_{B}\right)}^{T}.$$

Summarizing, the desired hyperplane be such that maximizes the distance between the means of each class and at the same time minimizes the variance in each class, leading to:

(9) $$\max_{\phi }\psi \left(\phi \right)=\max_{\phi }\frac{\phi^{T}S_{AB}\phi }{\phi^{T}S\phi }.$$

However, this optimization problem has an infinite number of solutions (for a solution $\phi^{*}$, all the vectors $c{\dot{\phi }}^{*}$ give exactly the same value). By replacing the denominator, without loss of generality, with an equality constraint to choose only one solution, the problem becomes:

(10) $$\begin{array}{c} \max_{\phi }\;\phi^{T}S_{AB}\phi , \end{array}$$

(11) $$\begin{array}{c} s.t.\;\phi^{T}S\phi =1\;, \end{array}$$

and the Lagrangian associated with this problem is:

(12) $$L_{LDA}\left(x,\lambda \right)=\phi^{T}S_{AB}\phi -\lambda \left(\phi^{T}S\phi -1\right)\;,$$

where λ is the Lagrange multiplier associated with the constraint defined in (11). The problem, being convex-since $S_{AB}$ is positive semidefinite-, the global minimum will be at the point for which:

(13) $$\frac{\delta L_{LDA}\left(x,\lambda \right)}{\delta x}=0\Leftrightarrow S_{AB}\phi -\lambda S\phi =0\;,$$

The optimal ϕ can be obtained as the eigenvector that corresponds to the smallest eigenvalue of the following generalized eigensystem [76]:

(14) $$S_{AB}\phi =\lambda S\phi .$$

For the case of a multiclass problem (as the one proposed in this study), the generalized problem becomes [76]:

(15) $$\begin{array}{c} S_{1,\ldots ,n}\phi =\lambda S\phi \;, \end{array}$$

(16) $$\begin{array}{c} S_{1,\ldots ,n}=\sum_{i=1}^{n}p_{i}\left(\bar{x_{i}}-\bar{x}\right){\left(\bar{x_{i}}-\bar{x}\right)}^{T}\;, \end{array}$$

(17) $$\begin{array}{c} S=S_{1}+S_{2}+\ldots +S_{n}\;, \end{array}$$

in which *n* is the number of classes. Then, the LDA can be used to identify the most significant features and their level of significance as expressed by the corresponding coefficient of the projection hyperplane. Each novel sample, once the correspondent original features set used in LDA is defined, can always be projected in the novel LDA-derived space, in order to compare it with previous samples already projected. Thus, in this particular problem, once all the original 45 features are gathered into a single one (derived from the LDA), it is always possible to convert a new sample defined by those 45 parameters into the correspondent LDA-derived single feature. Additionally, the LDA can include all the relevant information into N−1 features, in which *N* is the total number of original features included in the LDA, depending on the choice of the user. In this specific case, we chose to project all the original 45 features into a single one. However, due to intrinsic amplitude differences between features and in order to project them to the same values space range, a normalization procedure was applied to each sample of the dataset after applying the LDA. The samples mean value across each feature was subtracted to each data sample from that feature, and then divided by the corresponding feature standard deviation [44].

## 3. Results and Discussion

### 3.1. Optical Trapping

**Figure 8:**
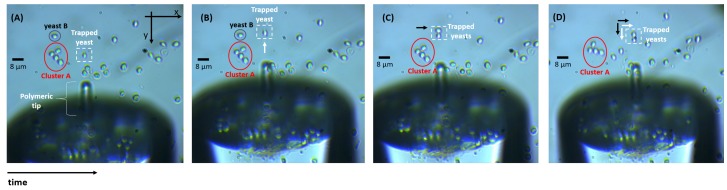
Snapshot sequence showing trapping and manipulation of living yeast cells using the polymeric tip. (**A**) A yeast cell is stably trapped in front of the tip. (**B**) The trapped yeast cell was moved along the *−y* direction—white arrow—(please consider cluster A as reference). (**C**) Yeast B moves to the right to the position of the polymeric lens beam focal spot (black arrow), being also trapped. (**D**) Both yeast cells are moved to the right (*+x* direction) and along the *+y* direction-white and black arrows by moving the polymeric optical fiber tip.

As previously reported in studies conducted by our lab [7,12,38,40], the polymeric spherical lenses fabricated through the guided photo-polymerization method described in Section 2.1.1 are able to stably trap and manipulate in 2D the three types of microparticles considered (8 μm PMMA spheres, 8 μm PS spheres and 6–7 μm living yeast cells). A sequence of video snapshots of trapping and manipulation of yeast cells using the lensed optical fiber tip is provided in Figure 8.

Observing the sequence of Figure 8, we can conclude that the fabricated tip is able to move one or more trapped yeasts along both *x* and *y* directions. The same outcome was observed for PMMA and PS microparticles. Thus, it was possible to immobilize each particle and acquire the back-scattered signal, ensuring that the radiation detected was derived exactly from that particle. For each test, the particle would be immobilized for period large enough to acquire a 120 s long data set.

### 3.2. Back-Scattered Signal Analysis

#### 3.2.1. Time Domain Features Analysis

**Figure 9:**
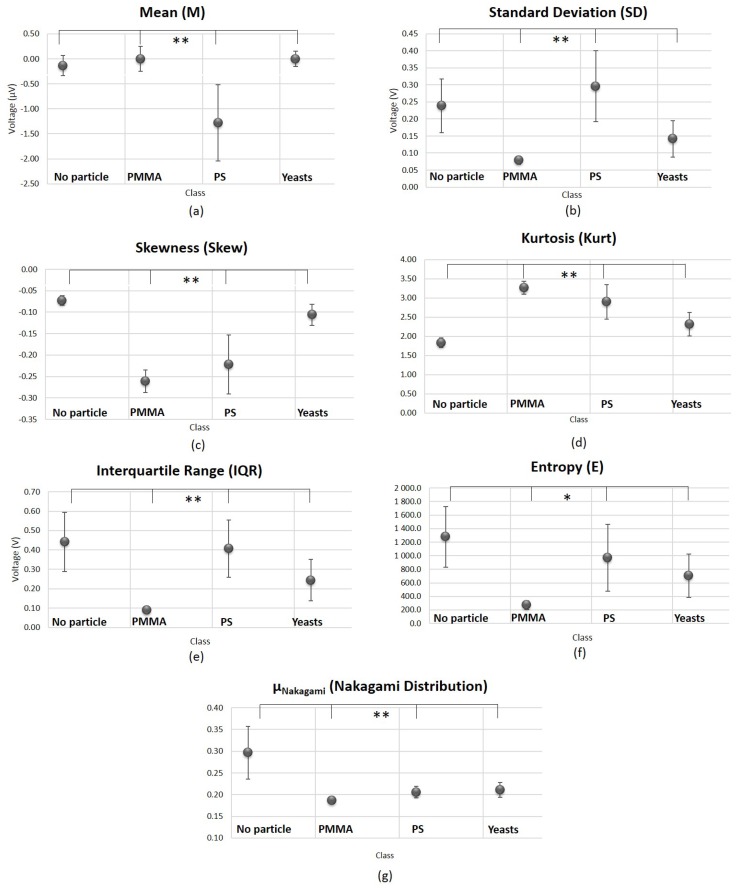
Graphical representation of the statistical comparisons relative to the back-scattered signal time-domain features analyzed (4 classes comparisons, Kruskal-Wallis test). (**a**–**f**) Comparisons made regarding time-domain statistics features. (**g**) Results obtained for $\mu_{Nakagami}$. The error bars represent standard error values. ** p<0.001. * p<0.05. $N_{No\;particle}=16$; $N_{PMMA}=16$; $N_{PS}=18$; $N_{Yeasts}=16$.

In Figure 9 are shown the results of the Kruskal-Wallis test performed in order to compare the 4 conditions (Class 1: “No particle”; Class 2: “PMMA”; Class 3: “PS” and Class 4: “Living yeast”). The Root Mean Square (RMS) was discarded from the original 45 set of features, for being highly correlated with Standard Deviation (SD) and, therefore, contributing with redundant information for the particles differentiation task. Regarding the statistics of the time-domain-graphics (a–f) from Figure 9, all the features revealed to be significantly different in statistical terms between the four conditions (*p*-value < 0.05; two-tailed). However, the Entropy showed to be less significant than the others, being the only one showing a *p*-value that, despite being lower than the significance level of 0.05, was higher than 0.001. Therefore, it is supposed to contribute with a significantly lower weight to the final single feature.

Relatively to the histogram-derived features, the $\omega_{Nakagami}$ was also discarded from the original pool of features, since it was highly affected by outliers. Due to its large variance, it could contribute with noise to the final single LDA-derived feature that we intended to obtain. On the contrary, $\mu_{Nakagami}$—Figure 9g—revealed to be suitable to distinguish the different particles type.

**Figure A1:**
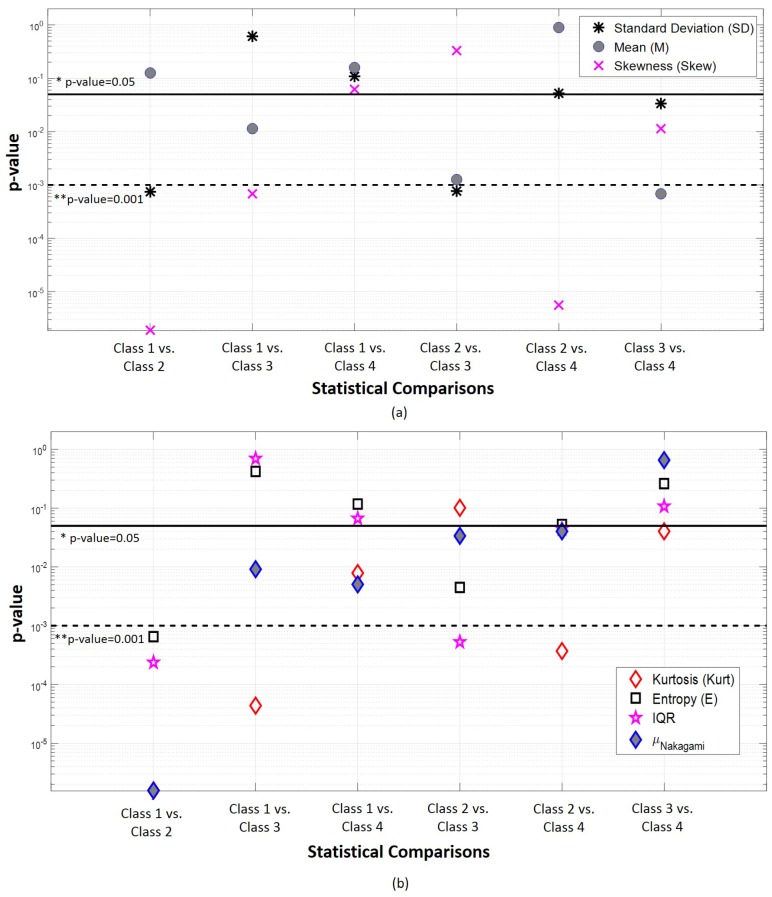
*p*-values obtained for the six post hoc parwise statistical comparisons (using the Mann Whitney test) that were performed after applying the Kruskal-Wallis test for 4 classes comparisons (time-domain features). (**a**) Statistical results obtained for Standard Deviation (SD), Mean (M) and Skewness (Skew). (**b**) *p*-values obtained for the comparisons relatively to Kurtosis (Kurt), Entropy (E), Interquartile Range (IQR) and $\mu_{Nakagami}$. Vertical axis is in logarithmic scale.

Post-hoc parwise statistical results for the seven time-domain features are presented in Figure A1, Appendix A. None of the analyzed features was able to differentiate with statistical significance all the binary classes combinations (for a level of 0.05). The results also reveal that, for all the features evaluated (excluding Kurtosis and $\mu_{Nakagami}$), it is difficult to distinguish Class 1 (“No particle”) from Class 4 (“Yeast cell”).

#### 3.2.2. Frequency Domain Features Analysis

#### Discrete Cosine Transform (DCT)-Derived Features

**Figure 10:**
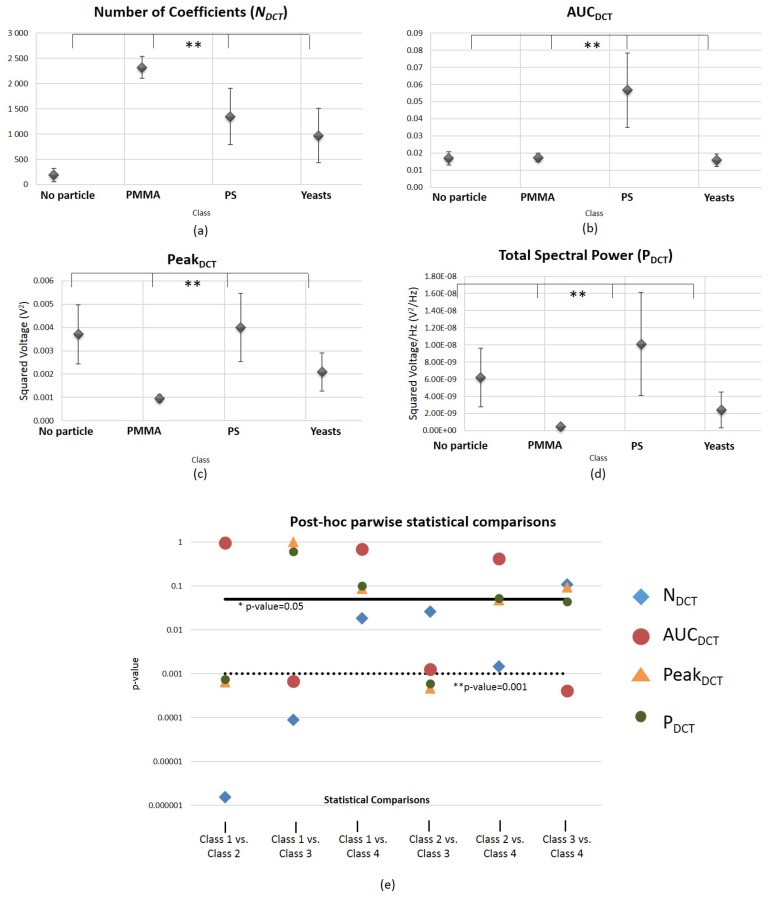
Graphical representation of the results obtained regarding the statistical comparisons performed using DCT-derived features for (**a**) Number of DCT coefficients that capture 98% of the total amount of energy of the original back-scattered signal (4 classes comparisons, Kruskal-Wallis test); (**b**) Total AUC of the DCT spectrum for the entire range of frequencies (4 classes comparisons, Kruskal-Wallis test); (**c**) Amplitude of the DCT spectrum peak for all range of frequencies analyzed (4 classes comparisons, Kruskal-Wallis test); (**d**) Total DCT spectral power (4 classes comparisons, Kruskal-Wallis test). The error bars represent standard error values. ** p<0.001. * p<0.05. (**e**) *p*-values obtained in the post hoc parwise statistical comparisons performed after Kruskal-Wallis for each one of the DCT-derived parameters evaluated, using the Mann Whitney test. Vertical axis is in logarithmic scale. Class 1—“no particle trapped”; Class 2—“PMMA particle trapped”; Class 3—”Polystyrene particle trapped”; Class 4—“Living yeast cell trapped”. $N_{No\;particle}=18$; $N_{PMMA}\;=\;16$; $N_{PS}=18$; $N_{Yeasts}=16$.

The results of Kruskal-Wallis (4 conditions) and Mann Whitney (2 conditions) tests obtained regarding the DCT-derived features $N_{DCT}$, $AUC_{DCT}$, $Peak_{DCT}$ and $P_{DCT}$ are presented in Figure 10. Graphics (a–d) reveal that $N_{DCT}$, $AUC_{DCT}$, $Peak_{DCT}$ and $P_{DCT}$ are significantly different between the 4 conditions analyzed with a significance level bellow 0.001, indicating that these are robust features for particles type differentiation. However, they follow a similar behaviour to the time-domain features relatively to the parwise comparisons. None of the four features are significantly different for all the binary combinations analyzed. The number of DCT coefficients that capture 98% of the total amount of energy of the original signal ($N_{DCT}$) showed to be the most robust feature, failing to be statistically significant only for the comparisons between Class 3 (“PS”) vs. Class 4 (“yeast cell”).

**Figure A2:**
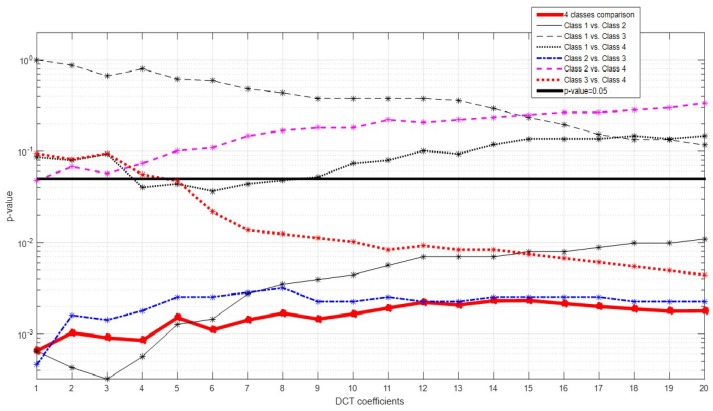
Graphical representation of *p*-values of the statistical comparisons regarding each coefficient extracted from the DCT applied to the original back-scattered signal. Vertical axis is in logarithmic scale. Class 1—“no particle trapped”; Class 2—“PMMA particle trapped"; Class 3—"Polystyrene particle trapped"; Class 4—“Living yeast cell trapped".

In Figure A2 from Appendix B is presented a graphic of *p*-values obtained for the Kruskal-Wallis test and the six parwise comparisons (Mann Whitney tests), for the 20 DCT coefficients extracted from the back-scattered signal. As well as the type of features analyzed so far, all 20 coefficients are able to statistically differentiate the four conditions. However, none of the 20 coefficients is suitable to distinguish Class 1 (“No particle”) versus Class 3 (“PS”). More details about the parwise comparisons are provided in Appendix B.

#### Wavelet Features

Similarly to time-domain and DCT, Wavelet-derived features allow particles type differentiation at the level of 4 classes comparisons (Table 4). However, contrary to the above features (time-domain and DCT-derived), there are four Wavelet-based features that, besides being suitable for 4 classes separation, also allow classes distinction in a parwise manner, for all the binary combinations considered. These are the features $E_{Haar}^{5}$ and $E_{Haar}^{6}$, representing the relative power of the 5th and 6th *Haar* Wavelet levels, respectively; and $E_{Db10}^{4}$ and $E_{Db10}^{5}$-relative power of the 4th and 5th level *Db10* Wavelet, respectively. Both types of Wavelet mother functions are then adequate for describing this type of signal in the frequency-domain. Additionally, this kind of features also reveal to have better discrimination capabilities between particles type, in comparison with the above features (time-domain and DCT parameters). It is expected that those four features would greatly contribute to the final LDA single feature. The adequacy of Wavelets to the targets scattered signals description task has been mentioned in previous studies [55,60,77], which reported that a few number of Wavelet coefficients is enough to reconstruct the original signal, once the mother wavelet is adequately chosen.

### 3.3. Towards a Single Feature for Particles Class Differentiation

**Figure 11:**
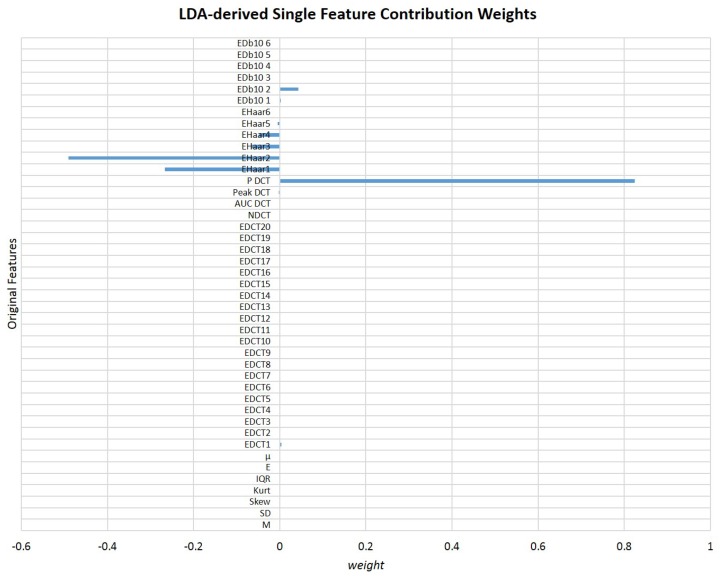
Graphical representation of the contribution weights of each one of the 43 original features for the final LDA-derived single feature (N = 43).

As concluded so far, despite of all the features analyzed (both in time- and frequency-domain) being suitable to differentiate the 4 classes considered, only a limited number is able to differentiate all pairs of classes. In fact, only four features derived from Wavelet are robust to both 4 classes and 2 classes comparisons. However, even if we intend to be selective in such a way to only consider those four features in the decision function, there are still four variables that must be considered, simultaneously. Considering that direct classification of particle type by “looking” simultaneously at four attributes is not a simple task, the calculation of a single feature that could be general enough to aid in that decision continues to seem viable. Taking into account that all the initial 43 features (corresponding to the 45 features excluding RMS and $\omega_{Nakagami}$) revealed to be useful, their information was considered in the LDA, since a linear projection of all of them could generate a suitable feature, although their individual contributions were not so robust for parwise comparisons. In Figure 11 is presented a graphic of the contribution weights of original features in the LDA-derived optimal final feature.

As expected, frequency-domain parameters contributed the most to the final feature. Previous studies have already reported that frequency-derived attributes of the back-scattered signal can provide valuable information about certain targets characteristics such as elasticity, geometrical shape, and size [55,56]. Additionally, the external modulation introduced in the laser light has probably strengthened even more the amount of relevant information carried by frequency-derived features. Particularly, according to LDA, the most significant feature is the total spectral power (for all the frequency range analyzed) obtained after applying the DCT. This is an interesting result, since, by analyzing separately each attribute, the Wavelet-derived features were the ones that showed more potential to differentiate particles type both in 4 classes and 2 classes comparisons. However, DCT-based parameters were already reported as relevant metrics to differentiate scatterers types in underwater conditions (for example, for fish species identification) [78]. Additionally, according to LDA, *Haar* mother Wavelet is more suitable than *Db10* in the differentiation task.

**Figure 12:**
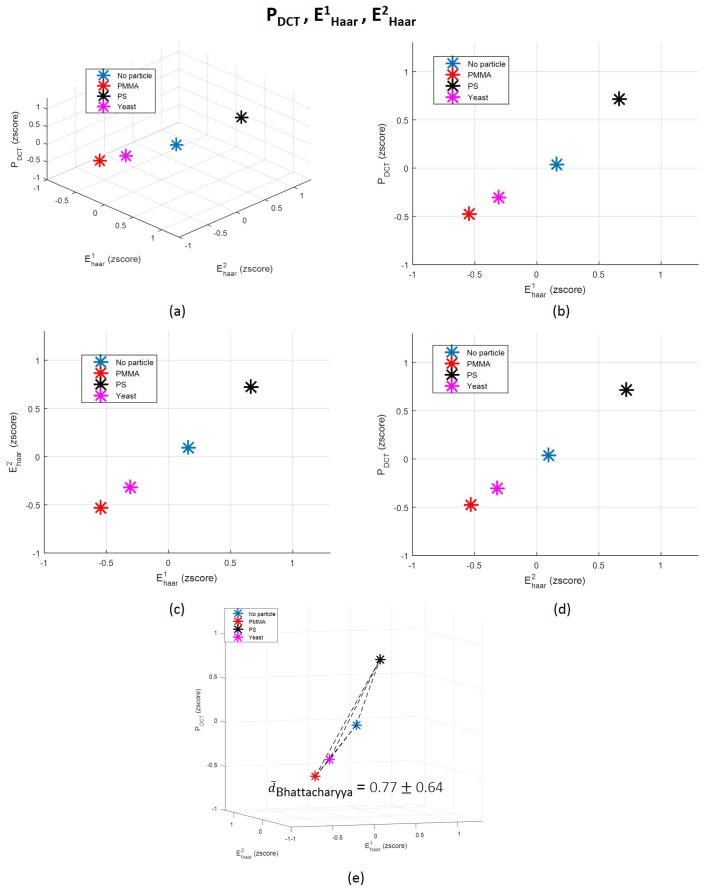
(**a**) 3-dimensional representation of the three original features ($P_{DCT}$, $E_{Haar}^{1}$ and $E_{Haar}^{2}$) which contributed the most for the final LDA-derived one; (**b**–**d**) 2D decompositions of the 3D space; and (**e**) correspondent average Bhattacharyya distance between each pair of classes distributions and graphical representation of each distance norm. Each point represents the mean value in the 3D space corresponding to each class: “Class 1: No particle”, “Class 2: PMMA”, “Class 3: PS” and “Class 4: Yeast”. $d_{Bhattacharyya}^{No\;particle/PMMA}=1.32$; $d_{Bhattacharyya}^{No\;particle/PS}=0.17$; $d_{Bhattacharyya}^{No\;particle/Yeast}=0.12$; $d_{Bhattacharyya}^{PMMA/PS}=1.67$; $d_{Bhattacharyya}^{PMMA/Yeast}=0.94$; $d_{Bhattacharyya}^{PS/Yeast}=0.43$.

**Figure 13:**
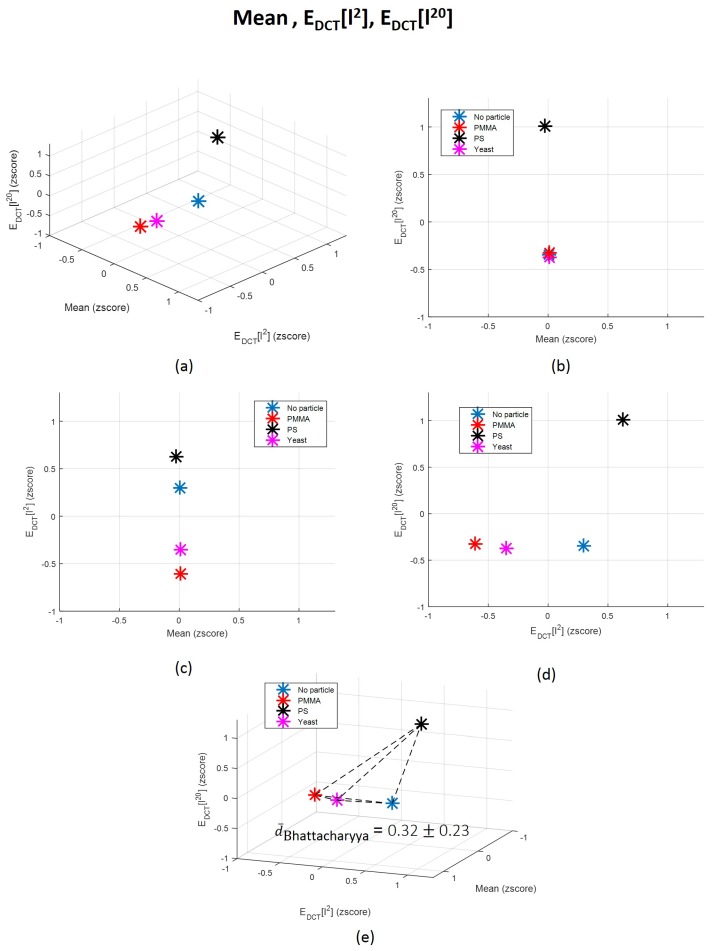
(**a**) 3-dimensional representation of the three original features (Mean, $E_{DCT}\left[l^{2}\right]$ and $E_{DCT}\left[l^{20}\right]$) whose information contributed the less for the final LDA-derived one; (**b**–**d**) 2D decompositions of the 3D space; and (**e**) correspondent average Bhattacharyya distance between each pair of classes distributions and graphical representation of each distance norm. Each point represents the mean value in the 3D space corresponding to each class: “Class 1: No particle”, “Class 2: PMMA”, “Class 3: PS” and “Class 4: Yeast”. $d_{Bhattacharyya}^{No\;particle/PMMA}=0.32$; $d_{Bhattacharyya}^{No\;particle/PS}=0.27$; $d_{Bhattacharyya}^{No\;particle/Yeast}=0.05$; $d_{Bhattacharyya}^{PMMA/PS}=0.73$; $d_{Bhattacharyya}^{PMMA/Yeast}=0.16$; $d_{Bhattacharyya}^{PS/Yeast}=0.38$.

The contribution of time-domain features is almost insignificant, revealing that they are likely noisy features. In fact, other studies that also used time-domain characteristics of the back-scattered signal arising from underwater targets reported that measures such as mean are not robust for targets differentiation, since it is highly dependent on changes on the target-sensor distance [51]. A study conducted in order to explain how dolphins recognize fishes according to their echoes also explored centralized measures not in the time-domain, but only after applying a Hilbert transform, enhancing the importance of the frequency-domain information rather than time-domain attributes [56]. However, it is important to refer that the behaviour of attributes can be different when used separately and when linearly projected in a novel parameter. Their behaviour could in fact change in order to be adequately fitted to the novel data distribution generated after features projection. Nevertheless, we decided to plot in a 3-dimensional space the three less significant and the three more important features according to the LDA, and to compute the average Bhattacharyya distance [79,80] between each pair of classes for each scenario. The corresponding graphics are in Figure 12 and Figure 13. The Bhattacharyya distance is a widely used metric in feature selection methods for Multivariate Data Analysis [79,80]. It is used to measure the separability of classes, being more robust than the widely known Mahalanobis distance [81], because it does not assume that the the two classes have equal or similar standard deviations. Since some of the features are highly variable, we decided to apply the Bhattacharyya distance to measure the mean separability degree between classes for the two scenarios described—Figure 12e and Figure 13e.

From graphics (e) from Figure 12 and Figure 13, we can observe that the mean Bhattacharyya distance between classes is higher considering the features space formed by the three most significant features according to LDA—$P_{DCT}$, $E_{Haar}^{1}$ and $E_{Haar}^{2}$—than by the three less relevant features (Mean, $E_{DCT}\left[l^{2}\right]$ and $E_{DCT}\left[l^{20}\right]$). This result shows that, as predicted by LDA, $P_{DCT}$, $E_{Haar}^{1}$ and $E_{Haar}^{2}$ are able to more efficiently separate the four classes in comparison with the other three attributes. This evidence also emphasizes, one more time, the importance of the frequency-domain characteristics. By observing individually the Bhattacharyya distance magnitude values corresponding to each pair of classes, it is possible to conclude that the classes that are more easily separable, for both case scenarios, are Class 2 and 3 (“PMMA” versus “PS”). This is an interesting conclusion, since these scatterers are exactly from the same size and shape, although from different types, which reinforces the robustness of the proposed method relatively to shape and size. In fact, we can say that particles are not differentiated by the amount of light they scatter due to their size, but, probably, due to the optical properties of its content (refractive index), as other attributes such as heterogeneity degree.

**Figure 14:**
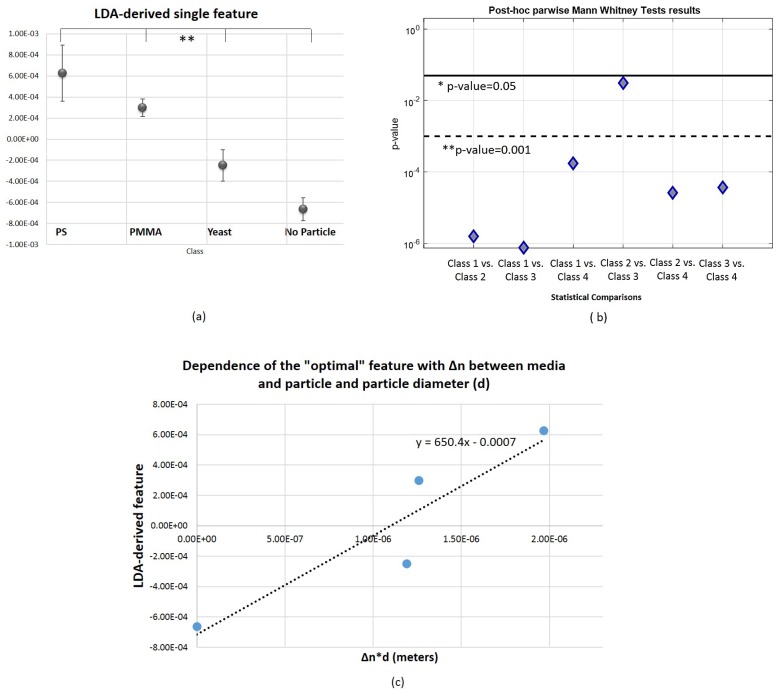
Statistical comparison between 4 classes and post-hoc parwise results for the obtained LDA-derived single feature and its dependence with Δn between media and each particle; and particle diameter (d). (**a**) Results of the Kruskal-Wallis test. Vertical axis is in logarithmic scale. ** p<0.001. * p<0.05. (**b**) Graphical representation of *p*-values obtained in the post-hoc parwise analysis using the Mann Whitney test. Vertical axis is in logarithmic scale. ** p<0.001. * p<0.05. (**c**) LDA-derived feature versus Δn·d, in which *d* is the particle diameter in meters, and corresponding fit line. *Spearman* correlation results (two-tailed): $r_{Spearman}=1.00$; $p_{Spearman}<0.05$.

After computing the optimal feature according to LDA, its statistical significance was analyzed to conclude if it would be suitable or not for differentiating particle types-Figure 14. The “optimal” feature showed to be statistically significant in the distinction of both 4 classes and in a parwise manner. In conclusion, this single feature was able to capture the most significant information from the original pool of 45 attributes. This novel attribute can therefore answer our research questions and turn them into a linearly separable problem. As it can be observed in Figure 14, the boundary values for this feature regarding each class are clearly delimited. Some studies have also mentioned that the aggregation of relevant features into a lower dimensional feature space could be advantageous in the context of target scattered signal analysis for scatterers differentiation [25,60].

Taking into account that the difference of the refractive index between the media and the scatterer (given by Δn) highly influences the amount of light scattered by a particle, as well as its size [82], we also investigated if there was a correlation between the novel “optimal” feature and these two parameters. Considering the graphic of Figure 14c, it is possible to observe a strong and statistically significant positive correlation between the computed LDA-derived feature and the product between Δn and particle diameter *d* ($r_{Spearman}=1.00$; $p_{Spearman}<0.05$). This result suggests that the higher the magnitude of this novel parameter, the higher the difference between the refractive index between the media and the particle and/or the higher is the diameter of the particle. Taking into account the results obtained above, we point out that this dependence is more likely due to the refractive index difference, since our distinction method showed to be robust in the differentiation task between PMMA and PS spheres of the same size. Ultimately, this novel parameter can be used to measure the refractive index or the size of the trapped particle. By finding such kind of calibration curve as the one depicted in Figure 14c, it could be possible to calculate the particle refractive index when its size is known or vice-versa, using only the information provided by the final “optimal” feature. Additionally, considering the dependence of the latter with the relative refractive index of the target, the proposed method could be also applied to the differentiation of *Malaria*-infected red blood cells in different infection stages. According to the study of Park et al. [36], healthy red blood cells and cells infected by *Malaria* are characterized by a different refractive index spatial distribution. Additionally, there are other parameters such as the nature and number of cell layers that define different cell types that can be translated by phase shifts on the back-scattered signal and therefore detected by the proposed method. However, all these possibilities must be further explored.

Considering that plasmonic tweezers provide simultaneous trapping and sensing similarly to the proposed method [19], we must to take into consideration the positive and negative aspects of this new technique relatively to plasmonics. In fact, although in-situ plasmonic sensing can give a precise measurement of the refractive index of trapped objects, its sensitivity to tiny refractive index changes of the surrounding environment can have a negative impact in trapping performance [22], as mentioned above. An unstable trapping of the scatterer can lead to the acquisition of noisy signals for sensing. In contrast, the polymeric lenses of the sensing platform proposed in this study ensure a stable trapping as already reported [7,12,41], and a clear differentiation of particle classes through the acquired back-scattered signal. Besides that, our method is not uniquely sensitive to a peak shift in a spectra (plasmonic resonance spectra). It is based on a multivariate feature allowing to differentiate particles that probably are not so easily distinguishable using refractive index measurements. However, plasmonic tweezers allow trapping of nanostructures, which is not possible with our technique. Our method is only able to trap particles with diameter above or approximately 1 μm [41]. Thus, an adaptation of the proposed method to a plasmonic tweezer could be a relevant contribution to literature, considering the advantages in terms of resolution and sensitivity of plasmonic tweezers and the simplicity of our method. However, we must also to consider the complexity of fabricating plasmonic structures.

One of the advantages of this method relatively to the other scattering techniques is related to the fact that particles differentiation is possible with the acquisition of back-scattered signal considering a single scatter angle [3,52]. It reveals to be advantageous also relatively to the methods which use image processing [83,84] instead of signal processing techniques for analyzing scattered light information. Scattered signal processing methods are associated with a lower computational cost, require less expensive equipment and allow the detection of objects with dimensions bellow the light diffraction limit, unlike imaging methods. However, the detection of such small particles using the proposed method must be adapted, since trapping using the proposed polymeric lenses is possible only for particles with diameter above or approximately 1 μm. Thus, the proposed method must be optimized to differentiate non-static particles with a diameter less than 1 μm (by including signal-derived features robust to particles motion); or additional modifications to the polymeric lens must be considered, such for example changes in the geometry of the lens or its patterning with a gold nanolayer to acquire plasmonic properties.

## 4. Conclusions

Along the present study, we performed an exploratory statistical analysis to select the back-scattered signal attributes most relevant for particles differentiation considering a four classes scenario: “No particle trapped”; “8 μm PMMA particle trapped”; “8 μm PS particle trapped” and “6–7 μm living yeast cell trapped”. We concluded that frequency-domain parameters such as Wavelet-derived features and attributes based on the Discrete Cosine Transform can provide valuable information about the scatterer type, in detriment of time-domain characteristics. Probably, the particle homogeneity degree (which is definitely different between synthetic or biological particles) contributes to differentiate PS and PMMA particles from living yeasts, and those differences were mirrored by frequency-domain signal differences.

Considering that a single parameter could facilitate results interpretation by any kind of interrogation system, we also tried to fuse 45 relevant signal-derived attributes into a single variable, using the Linear Discriminant Analysis (LDA). This novel feature was successful in differentiating the four classes considered, both in a 4 classes and parwise manner. We also found that, there was a strong and significant correlation between this “optimal” feature and the difference between the refractive index of the media and the scatterer and its diameter. This suggests that, as expected, the relation between particle diameter and its relative refractive index regarding to media are relevant differentiation parameters. That influence might be reflected in the frequency component of the scattered signal, due to the differences in the light optical path considering different scatterers in the same media. Probably, the signal frequency-domain parameters showed to be more relevant than the time-domain also because they were able to more efficiently transduce this dependence than the latter.

However, the proposed method has also limitations. More complex differentiation problems, involving for example distinct biological cell types, may not be linearly separable. In these cases it might be necessary to apply more complex statistical methods. Nevertheless, the present study can be a valuable contribution to literature, making available an extensive battery of features that can be applied in such complex methods.

In conclusion, this single parameter can contribute for the development of a low-cost and simple method for particles differentiation using OT to immobilize the targets and to further analyze the back-scattered signal arising from the trapped particle. Since the particle remains immobilized during measurements in front of the probe, it can be highly robust. Additionally, it does not require bulky equipment, fluorescent probes or antibodies, being mainly characterized by a flexible and biocompatible spherical lens on the top of an optical fiber and a photodetector. Such kind of method can be very useful for cell and other microparticles differentiation with applications in Medicine (e.g., differentiation between healthy and infected cells or live and death cells).

## Figures and Tables

**Table 1:** Optical and morphological characteristics of the particle samples used in the experiment. RI-Refractive Index. Nr.—Number.

Solution	Solvent	Particles Type	Particles Diameter (d)	Particles RI	Nr. of Particles Used
1	De-ionized water (*n* = 1.327)	Polystyrene microspheres	8 μm	1.5731 [42]	18
2		PMMA microspheres	8 μm	1.4843 [42]	16
3		Living yeast cells	6–7 μm	1.49–1.53 [43]	16

**Table 2:** Dataset characterization. Each sample/instance corresponds to each 2 s back-scattered signal. In the case of class 1/“no particle” condition, each particle ID corresponds to different acquisitions made with no particle in front of the fiber tip. Nr—number. Avg—average.

Class	1: “No Particle”	2: “PMMA Particle”	3: “PS Particle”	4: “Living Yeast Cell”
Nr. of Particles	16	16	18	16
Avg. nr. of signal portions per particle	59 ± 0	57 ± 2	54 ± 5	59 ± 2
Total nr. of signal portions (all particles)	949	919	971	939

**Table 3:** Table summarizing the back-scattered signal features/parameters set evaluated in this study.

Type	Group	Number	Feature/Parameter
Time Domain	Time Domain Statistics	1	Mean (M)
		2	Standard Deviation (SD)
		3	Root Mean Square (RMS)
		4	Skewness (Skew)
		5	Kurtosis (Kurt)
		6	Interquartile Range (IQR)
		7	Entropy ( E )
	Time Domain Histogram	8	$\mu_{Nakagami}$
		9	$\omega_{Nakagami}$
Frequency Domain	Discrete Cosine Transform (DCT)	10	1st Coefficient ($E_{DCT}\left[l^{1}\right]$)
		11	2nd Coefficient ($E_{DCT}\left[l^{2}\right]$)
		12	3rd Coefficient ($E_{DCT}\left[l^{3}\right]$)
		13	4th Coefficient ($E_{DCT}\left[l^{4}\right]$)
		14	5th Coefficient ($E_{DCT}\left[l^{5}\right]$)
		15	6th Coefficient ($E_{DCT}\left[l^{6}\right]$)
		16	7th Coefficient ($E_{DCT}\left[l^{7}\right]$)
		17	8th Coefficient ($E_{DCT}\left[l^{8}\right]$)
		18	9th Coefficient ($E_{DCT}\left[l^{9}\right]$)
		19	10th Coefficient ($E_{DCT}\left[l^{10}\right]$)
		20	11th Coefficient ($E_{DCT}\left[l^{11}\right]$)
		21	12th Coefficient ($E_{DCT}\left[l^{12}\right]$)
		22	13th Coefficient ($E_{DCT}\left[l^{13}\right]$)
		23	14th Coefficient ($E_{DCT}\left[l^{14}\right]$)
		24	15th Coefficient ($E_{DCT}\left[l^{15}\right]$)
		25	16th Coefficient ($E_{DCT}\left[l^{16}\right]$)
		26	17th Coefficient ($E_{DCT}\left[l^{17}\right]$)
		27	18th Coefficient ($E_{DCT}\left[l^{18}\right]$)
		28	19th Coefficient ($E_{DCT}\left[l^{19}\right]$)
		29	20th Coefficient ($E_{DCT}\left[l^{20}\right]$)
		30	Number of coefficients that capture 98% of the original signal ($N_{DCT}$)
		31	Total spectrum Area Under Curve (AUC) ($AUC_{DCT}$)
		32	Maximum peak amplitude ($Peak_{DCT}$)
		33	Total spectral power ($P_{DCT}$)
	Wavelet Packet Decomposition	34	Haar Relative Power 1st level ($E_{Haar}^{1}$)
		35	Haar Relative Power 2nd level ($E_{Haar}^{2}$)
		36	Haar Relative Power 3rd level ($E_{Haar}^{3}$)
		37	Haar Relative Power 4th level ($E_{Haar}^{4}$)
		38	Haar Relative Power 5th level ($E_{Haar}^{5}$)
		39	Haar Relative Power 6th level ($E_{Haar}^{6}$)
		40	Db10 Relative Power 1st level ($E_{Db10}^{1}$)
		41	Db10 Relative Power 2nd level ($E_{Db10}^{2}$)
		42	Db10 Relative Power 3rd level ($E_{Db10}^{3}$)
		43	Db10 Relative Power 4th level ($E_{Db10}^{4}$)
		44	Db10 Relative Power 5th level ($E_{Db10}^{5}$)
		45	Db10 Relative Power 6th level ($E_{Db10}^{6}$)

**Table 4:** Table presenting *p*-value results of the statistical comparisons regarding 4 classes and post-hoc parwise comparisons for Wavelet-derived features. ** p<0.001. * p<0.05. Blank cells represent comparisons which were not statistically significant for a level of 0.05. Class 1—“no particle trapped”; Class 2—“PMMA particle trapped”; Class 3—“Polystyrene particle trapped”; Class 4—“Yeast cell trapped”.

Type of Wavelet	Wavelet Energy Level	4 Classes Comparisons	Class 1 vs. Class 2	Class 1 vs. Class 3	Class 1 vs. Class 4	Class 2 vs. Class 3	Class 2 vs. Class 4	Class 3 vs. Class 4
Haar	1st	**	**			**		*
	2nd	**	**			**		*
	3rd	**	*			**		*
	4th	**	*			**		*
	5th	**	**	**	**	*	**	**
	6th	**	**	**	*	*	**	**
Db10	1st	**	**			**		*
	2nd	**	**			**		
	3rd	**		**		**		**
	4th	**	**	**	*	*	**	**
	5th	**	**	**	**	*	**	**
	6th	**	*	**		*	*	**

## Appendix B. Frequency Domain Features Analysis-Discrete Cosine Transform (DCT) Coefficients

In Figure A2 is presented a graphic of *p*-values obtained for the Kruskal-Wallis test and the six parwise comparisons (Mann Whitney tests), for the 20 DCT coefficients extracted from the back-scattered signal. All the 20 coefficients are able to statistically differentiate the four conditions. However, none of the 20 coefficients is suitable to distinguish Class 1 (“No particle”) versus Class 3 (“PS”), which can be successfully made using some of the time-domain and other type of DCT-derived features ($N_{DCT}$ and $AUC_{DCT}$). In contrast, all 20 coefficients can significantly differentiate Class 1 from Class 2 (“No Particle” and “PS particle”, respectively) and Class 2 (“PMMA”) versus Class 3 (“PS”). Additionally, the fifth, sixth, seventh and eighth coefficients are the most robust parameters, being successful in four of six binary problems. This effect was already expected since as higher the order of the DCT coefficient-which were ordered according to its magnitude, from the highest to the lowest-the less amount of novel information is added about the original signal. Thus, it was expected that the information carried by higher order coefficients would lose significance.

## Abbreviations

The following abbreviations are used in this manuscript:

AUC	Area Under the Curve
CMOS	Complementary Metal-Oxide-Semiconductor
COTs	Conventional Optical Tweezers
DAQ	Data Acquisition Board
DCT	Discrete Cosine Transform
E	Entropy
FFT	Fast-Fourier Transform
IQR	Interquartile Range
IoT	Internet of Things
Kurt	Kurtosis
LDA	Linear Discriminant Analysis
M	Mean
OFT	Optical Fiber Tweezers
OT	Optical Tweezers
PBS	Phosphate Buffered Saline
PCA	Principal Component Analysis
PDF	Probability Density Function
PMMA	Poly(methyl methacrylate)
PS	Polystyrene
RBC	Red Blood Cell
RI	Refractive Index
RMS	Root Mean Square
SD	Standard Deviation
Skew	Skewness
SMF	Single Mode Fiber
SNR	Signal-to-noise Ratio
